# Prognostic significance of beta‐adrenergic receptor expression in oesophageal adenocarcinoma

**DOI:** 10.1002/2056-4538.70070

**Published:** 2026-01-04

**Authors:** Talita Oliveira, Swathi Sasi, James Trainor, Damian T McManus, Stephen McQuaid, Claire Lewis, Jacqueline A James, Helen G Coleman, Úna C McMenamin, Richard C Turkington

**Affiliations:** ^1^ Patrick G. Johnston Centre for Cancer Research Queen's University Belfast Belfast UK; ^2^ Department of Pathology Belfast Health and Social Care Trust Belfast UK; ^3^ Northern Ireland Biobank, The Patrick G. Johnston Centre for Cancer Research Queen's University Belfast Belfast UK; ^4^ Centre for Public Health Queen's University Belfast Belfast UK

**Keywords:** oesophageal cancer, adenocarcinoma, beta adrenergic receptors, tumour biomarker, immunohistochemistry

## Abstract

Expression of the β‐adrenergic receptors' family has been associated with survival outcomes in multiple different cancer types, showing their potential to act as prognostic factors. No previous work has evaluated these receptors in relation to survival in oesophageal adenocarcinoma. We sought to analyse the expression of β_1_ and β_2_ adrenergic receptors in oesophageal adenocarcinoma and their association with survival outcomes. The expression of β_1_ and β_2_ adrenergic receptors was evaluated in a cohort of oesophageal adenocarcinoma patients treated with neo‐adjuvant chemotherapy prior to surgical resection at the Northern Ireland Cancer Centre between 2004 and 2012. Immunohistochemical staining for was assessed using a Tissue Microarray with triplicate tumour cores. Cox proportional hazards regression was used to investigate the association of β_1_ and β_2_ adrenergic receptor expression with survival outcomes, including adjustment for clinical factors. In total, 115 and 122 patients were assessed for β_1_ and β_2_ adrenergic receptor expression, respectively. In adjusted analysis, high β_2_ adrenergic receptor expression was associated with improved recurrence‐free [hazard ratio [HR] 0.57, 95% CI 0.33–0.97] and overall survival (HR 0.53, 95% CI 0.30–0.94) with restriction to gastro‐oesophageal junction tumours showing a stronger association with improved overall survival (HR 0.27, 95% CI 0.13–0.59). No significant association was observed for β_1_ adrenergic receptor expression and any survival outcome. In summary, we found that higher expression of the β_2_ adrenergic receptor was associated with a significant improvement in survival in oesophageal adenocarcinoma patients, and gastro‐oesophageal junction tumours in particular, treated with neoadjuvant chemotherapy followed by surgical resection.

## Introduction

The incidence of oesophageal adenocarcinoma (OAC) has risen rapidly in the past four decades in western countries but 5‐year survival rates remain low at less than 21% [1, 2]. For earlier stage, resectable tumours, even with improvements in neoadjuvant therapeutic strategies and surgical resection techniques, survival remains poor at 35–45% with a lack of molecular tools for patient stratification and estimation of OAC patient prognosis [3, 4].

The beta‐adrenergic receptors (βARs) are a family of G protein‐coupled receptors ubiquitously expressed in mammalian cells and play an important role in several physiological processes, including smooth muscle contraction [5]. Both β_1_ and β_2_ adrenergic receptors (β_1_AR, β_2_AR) are widely targeted by β‐blockers to treat conditions such as hypertension or cardiac arrhythmia, and more recently, growing evidence has supported the antitumor effect of β‐blockers for some cancer types [6, 7].

Previous reports have shown that βARs are associated with cancer survival outcomes [8, 9, 10, 11, 12]. β_2_AR expression in 147 surgically resected colorectal cancers was associated with worse overall survival (HR 2.27; 95% CI 1.51–3.72; *p* < 0.01) than β_2_AR negative tumours [8] and, in 100 gastric tumours, above‐median β_2_AR expression was associated with increased tumour size, lymph node positivity and poor differentiation [9]. Recent evidence has also implicated β_1_AR as a mediator of CD8+ T cell exhaustion and, following activation by the sympathetic nervous system, may directly regulate T cell effector functions and immune response in tumours [13]. To date, neither β_1_AR nor β_2_AR has been assessed in OAC.

The aim of this study was to investigate the expression of β_1_‐ and β_2_AR in OAC patients treated with neo‐adjuvant chemotherapy followed by surgical resection and to determine the associations between βAR expression and survival outcomes. The identification of additional prognostic factors following radical treatment could inform surveillance strategies and select patients for more intensive follow‐up.

## Materials and methods

### Study design and cohort

Patients (*n* = 152) with OAC who had undergone neoadjuvant chemotherapy followed by surgery between 2004 and 2012 at the Northern Ireland Cancer Centre were identified and a tissue microarray (TMA) was constructed as previously described [14]. The archived tissue resection specimens were retrieved by the Northern Ireland Biobank, which has overarching ethics approval for consent, collection, storage and distribution of human tissue samples and associated data (OREC reference 16/NI/0030), enabling researchers to use samples from prospective and retrospective collections [15]. All patients received neo‐adjuvant chemotherapy prior to surgical resection and had a median follow‐up time of 48.8 months. Pathological staging was defined according to International Union Against Cancer (UICC) TNM staging, 8th edition. Eight specimens had no tumour tissue identifiable and 3 patients died within 3 months of surgery of causes unrelated to their cancer, resulting in 141 cases entering the final analysis. This project was approved by the Northern Ireland Biobank (NIB15‐0176). The study was conducted in accordance with the Reporting Recommendations for Tumour Marker Prognostic Studies (REMARK) criteria (supplementary material, Table S1), the International Conference of Harmonisation of Good Clinical Practice and the Declaration of Helsinki [16].

### Immunohistochemistry

Immunohistochemistry (IHC) analysis was performed in the Precision Medicine Centre of Excellence at Queens University Belfast. TMA sections of 3 μm were deparaffinised and endogenous peroxidase activity was quenched with 0.3% hydrogen peroxide prior to drying overnight at 37°C. Slide labelling and staining was performed using the Ventana Discovery XT automated immunostainer (Ventana Medical Systems Inc., Tucson, AZ, USA). Antibodies to β_1_AR (ab3442, Abcam, Cambridge, UK) and β_2_AR (ab182136, Abcam, Cambridge, UK) were used according to the manufacturer's instructions. All sections were scanned on an Aperio AT2 scanner and viewed as digital images on Xplore (PathXL).

### Scoring methods

Two independent observers (SS and JT), who were blinded to the clinical data, reviewed and scored the TMAs. In case of discordance between observers, a consensus score was agreed after deliberation. The percentage of tumour cells staining positive was assessed, and the intensity was evaluated based on the degree of cytoplasmic staining. The scoring range for intensity was between 0 and 3, in which 0 = staining was not observed, 1 = weak, 2 = moderate, and 3 = strong staining.

A score for β_1_‐ and β_2_AR was calculated by multiplying the values for intensity and percentage of tumour cells to obtain a value between 0 and 300. The overall intensity for the whole core was assessed and multiplied by the proportion of tumour cells stained in divisions of 10.

### Clinical variables

Patients' clinical and pathological data was retrieved at the Northern Ireland Cancer Centre. Tumour location was divided into lower third of oesophagus [greater than 5 cm proximal to the gastro‐oesophageal junction (GOJ)], Siewert 1 (within 1–5 cm above the GOJ), Siewert 2 (within 1 cm above and 2 cm below the GOJ) and Siewert 3 (2–5 cm below the GOJ) [17, 18]. Involvement of the circumferential resection margin (CRM) was defined as tumour cells at, or within 1 mm, of the resection margin [19]. Response to chemotherapy on positron emission tomography (PET) scan was defined as a reduction in the tumour glucose standard uptake value (SUV) of 35% or more [20]. Pathological response was assessed in the matched resection specimens according to the method described by Mandard *et al* with a responder defined as Tumour Regression Grade (TRG) ≤2 [21, 22].

### Statistical analysis

Maximum scores from triplicate cores for each patient were used to classify patients according to β_1_‐ and β_2_AR expression. The median value of the cohort's overall maximum score was selected as a cut‐off value to define β_1_‐ and β_2_AR low and high expression, with partitioning of the data according to tertiles also explored.

Chi‐square tests were used to compare tumour characteristics and patient demographics, according to β_1_‐ and β_2_AR median expression status. Associations between βAR expression and recurrence‐free, overall and cancer‐specific survival were investigated using Cox proportional hazards regression producing unadjusted and adjusted hazard ratios (HRs) and 95% confidence intervals (CIs). Patients were followed from the date of OAC diagnosis until their last review appointment, date of recurrence, date of death or end of study follow‐up. All analyses were adjusted for age at diagnosis, sex, pathological nodal stage, grade, primary tumour location (lower third or GOJ), lymphatic vascular invasion, CRM, PET response and smoking. Sensitivity analysis was conducted in which the study cohort was restricted to patients diagnosed with GOJ tumours. Analysis was undertaken using STATA 14 software (College Station, TX, USA).

## Results

### Patient demographics and immunohistochemical staining

A total of 141 patients with OAC who had undergone platinum‐based neoadjuvant chemotherapy followed by surgery between 2004 and 2012 in Northern Ireland were included. Of these, 130 were analysed following the exclusion of 5 cases with no clinical information, 5 cases who had complete pathological response, and 1 patient who had metastases present at the time of surgery (supplementary material, Figure S1). Cases that had no tumour in all three cores for β_1_AR (*n* = 15) and β_2_AR (*n* = 8) were also excluded. The majority of included patients were males (76%) and the most common primary tumour location was the GOJ (83%) (Table 1 and supplementary material, Table S2).

**Table 1:** Characteristics of OAC patients by β_1_ and β_2_AR expression

	β_1_AR status (*n* = 115)			β_2_AR status (*n* = 122)		
	Low (*n* = 40)	High (*n* = 75)	*p*	Low (*n* = 56)	High (*n* = 66)	*p* (chi‐squared)
Sex						
Male	30 (75.0)	57 (76.0)	0.91	41 (73.2)	51 (77.3)	0.60
Female	10 (25.0)	18 (24.0)		15 (26.8)	15 (22.7)	
Age at diagnosis (years)						
<50	3 (7.5)	7 (9.3)	0.48	7 (12.5)	4 (6.1)	0.21
50–59	12 (30.0)	13 (17.4)		14 (25.0)	11 (16.7)	
60–69	17 (42.5)	37 (49.3)		26 (46.4)	32 (48.5)	
≥70	8 (20.0)	18 (24.0)		9 (16.1)	19 (28.7)	
Smoking status						
Non‐smoker	14 (35.0)	15 (20.0)	0.17	11 (19.6)	18 (27.3)	0.45
Ex‐smoker	12 (30.0)	35 (46.7)		27 (48.2)	23 (34.9)	
Current smoker	8 (20.0)	18 (24.0)		13 (23.3)	16 (24.2)	
Unknown	6 (15.0)	7 (9.3)		5 (8.9)	9 (13.6)	
Alcohol						
Non‐drinker	17 (42.5)	23 (30.7)	0.17	14 (25.0)	27 (40.9)	0.11
Drinker	15 (37.5)	42 (56.0)		34 (60.7)	28 (42.4)	
Unknown	8 (20.0)	10 (13.3)		8 (14.3)	11 (16.7)	
Primary tumour site						
Lower third	8 (20.0)	10 (13.3)	0.35	8 (14.3)	12 (18.2)	0.56
Gastro‐oesophageal junction	32 (80.0)	65 (86.7)		48 (85.7)	54 (81.8)	
Siewert classification*						
Siewert I	21 (65.6)	43 (66.2)	0.96	32 (66.7)	32 (59.3)	0.44
Siewert II/III	11 (34.4)	22 (33.8)		16 (33.3)	22 (40.7)	
PET response						
No	13 (32.5)	29 (38.7)	0.74	18 (32.1)	24 (36.4)	0.78
Yes	20 (50.0)	36 (48.0)		28 (50.0)	33 (50.0)	
Unknown	7 (17.5)	10 (13.3)		10 (17.9)	9 (13.6)	
Lymphatic vascular invasion						
No	10 (25.0)	28 (37.3)	0.18	16 (28.6)	25 (37.9)	0.34
Yes	29 (72.5)	47 (62.7)		40 (71.4)	40 (60.6)	
Unknown	1 (2.5)	0 (0.0)		0	1 (1.5)	
Grade						
Well or moderate	18 (45)	30 (40)	0.61	18 (32.1)	35 (53.0)	0.02
Poor	22 (55)	45 (60)		38 (67.9)	31 (47.0)	
Circumferential resection margin status						
Negative	26 (65.0)	38 (50.7)	0.29	26 (46.4)	42 (63.6)	0.11
Positive	14 (35.0)	36 (48.0)		29 (51.8)	24 (36.4)	
Unknown	0 (0.0)	1 (1.3)		1 (1.8)	0	
Surgical T stage						
1	1 (2.5)	11 (14.7)	0.06	2 (3.6)	10 (15.2)	0.18
2	9 (22.5)	15 (20.0)		12 (21.4)	13 (19.7)	
3	30 (75.0)	44 (58.7)		39 (69.6)	41 (62.1)	
4	0 (0.0)	5 (6.6)		3 (5.4)	2 (3.0)	
Surgical N stage						
0	13 (32.5)	29 (38.6)	0.92	17 (30.4)	26 (39.4)	0.31
1	8 (20.0)	14 (18.7)		15 (26.8)	9 (13.6)	
2	9 (22.5)	14 (18.7)		12 (21.4)	14 (21.2)	
3	10 (25.0)	18 (24.0)		12 (21.4)	17 (25.8)	

* Restricted to patients with gastro‐oesophageal junction tumours, T = tumour, N = nodal.

**Figure 1:**
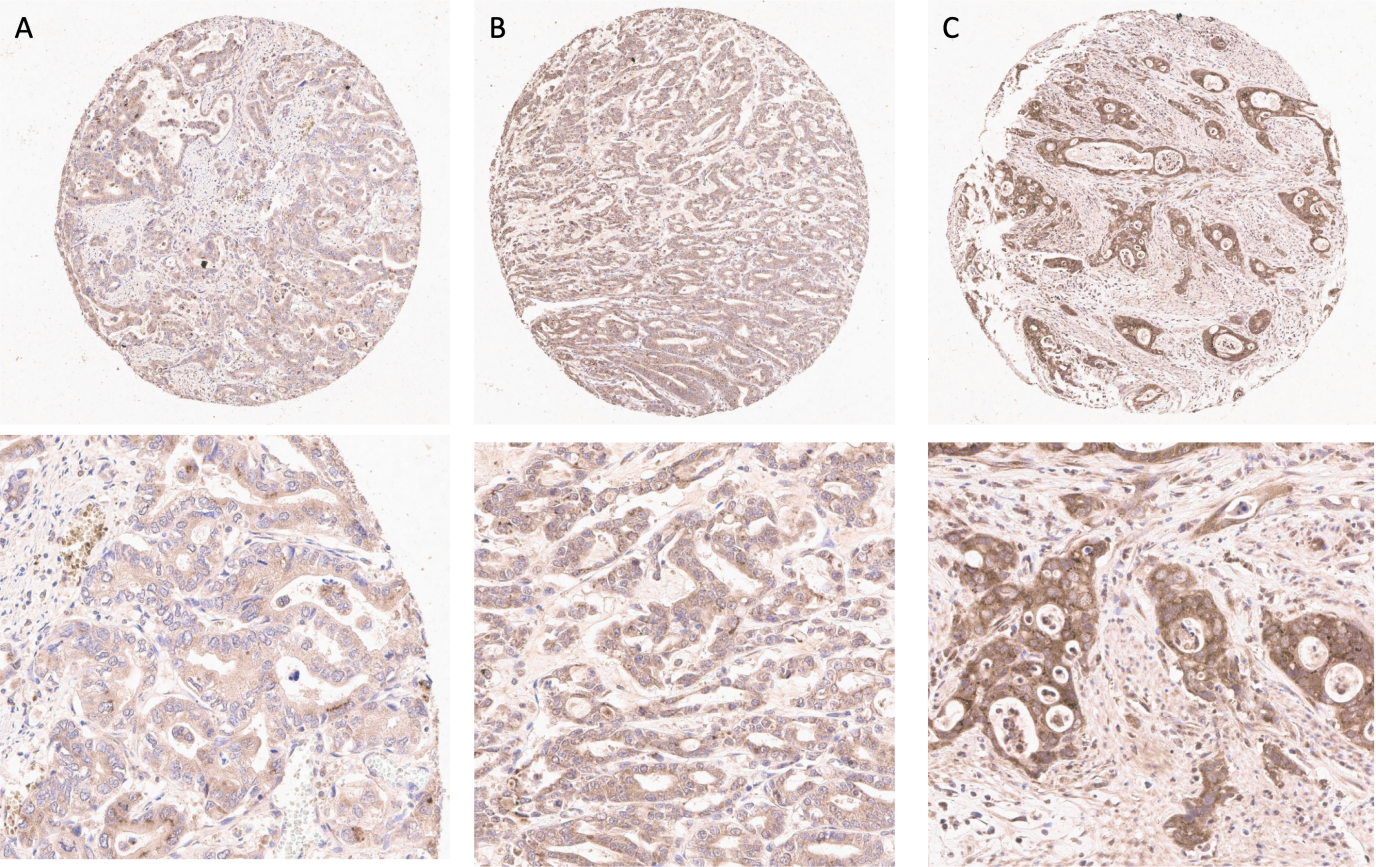
β_1_AR Immunohistochemical staining. Representative overview (×10) and detail (×40) views of tissue microarray tumour cores showing (A) weak (score 40), (B) moderate (score 160), and (C) strong (score 300) staining for β_1_AR.

**Figure 2:**
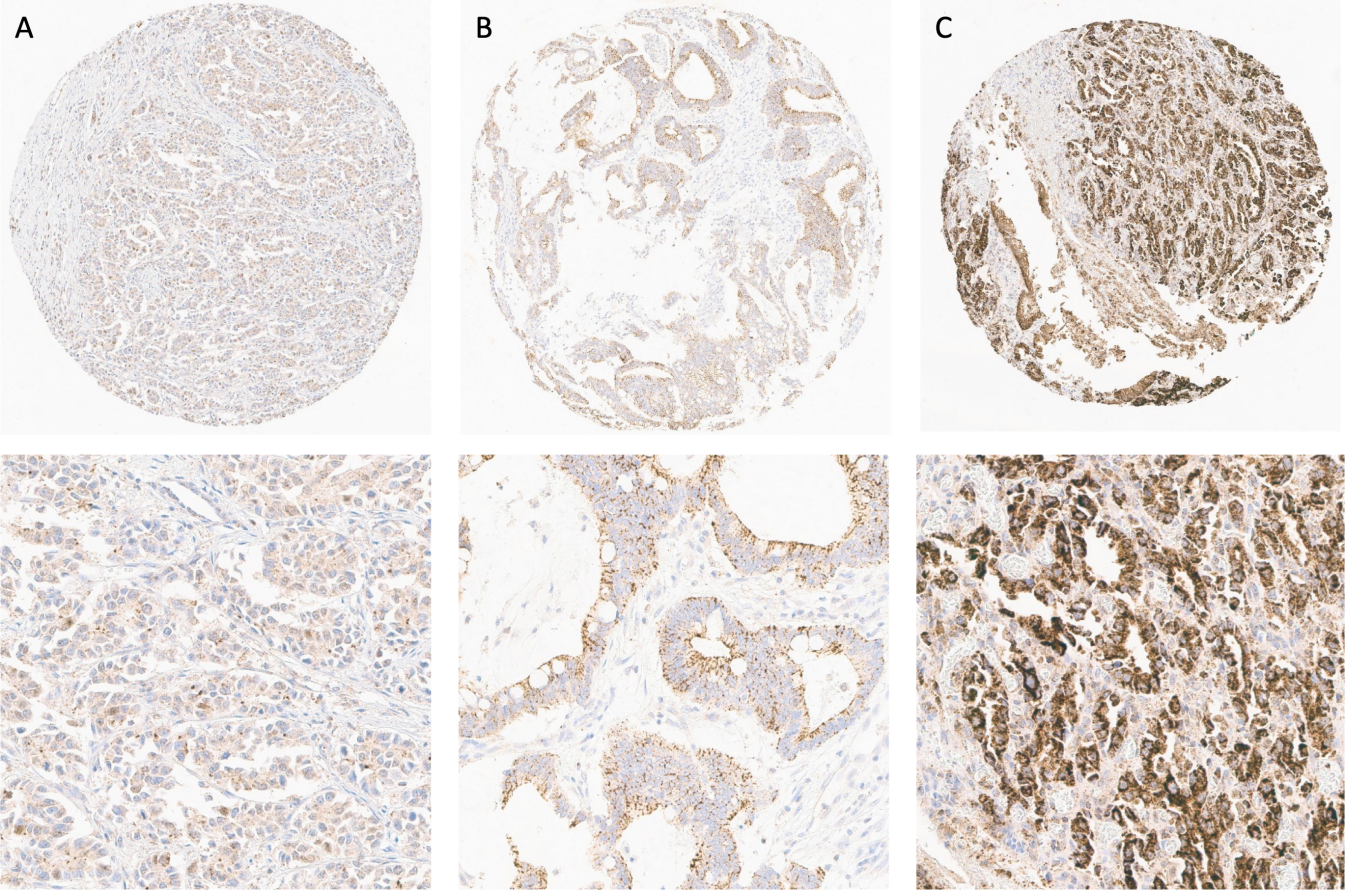
β_2_AR Immunohistochemical staining. Representative overview (×10) and detail (×40) views of tissue microarray tumour cores showing (A) weak (score 30), (B) moderate (score 180), and (C) strong (score 300) staining for β_2_AR.

Expression of β_1_AR and β_2_AR was localised predominantly in the cytoplasm and at the cell membrane of OAC cells (Figures 1 and 2). No expression of β_1_AR and β_2_AR was observed in the normal squamous epithelial lining of the oesophagus.

The median scores for β_1_AR and β_2_AR were 270 and 180, respectively, and these were used to dichotomise patients into high and low expression groups (supplementary material, Figure S2). No significant differences in the clinicopathological characteristics were observed between high and low β_1_AR and β_2_AR expressing patients with the exception of tumour grade (Table 1). The β_2_AR low group demonstrated a significantly greater percentage of poorly differentiated tumours when compared to the β_2_AR high group (67.9 versus 47%; *p* < 0.02).

### β _1_AR/β_2_AR expression and survival outcomes

In the unadjusted, adjusted and Kaplan Meier analyses, the expression of β_1_AR had no association with recurrence‐free, overall or cancer‐specific survival (supplementary material, Figure S3 and Table S3).

**Figure 3:**
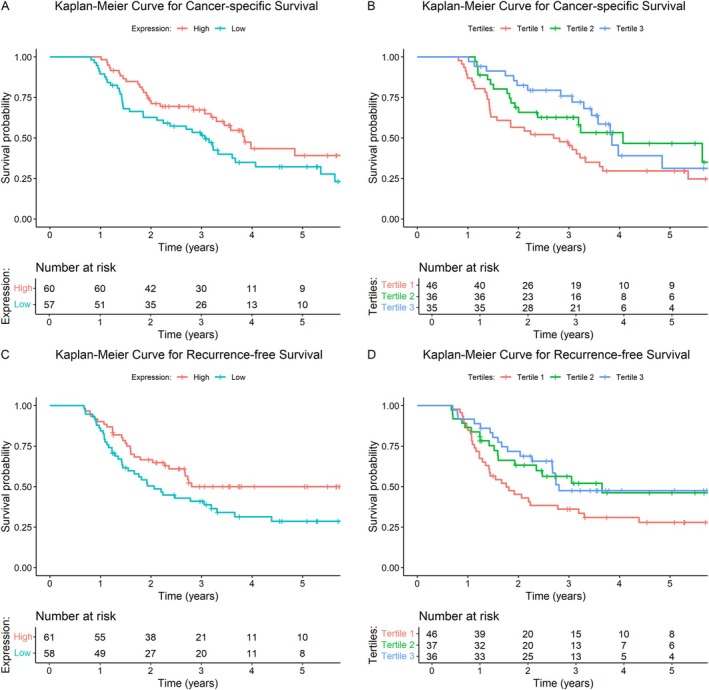
Kaplan–Meier survival analysis of β_2_AR expression for: (A) cancer‐specific survival estimates by high and low β_2_AR expression (median cut‐off value = 180); (B) cancer‐specific survival by tertiles (tertile 1 <140; tertile 2140–270; tertile 3 >270); (C) recurrence‐free survival by high and low β_2_AR expression; and (D) recurrence‐free survival estimates by tertiles.

For β_2_AR, the unadjusted analysis showed no significant association with any survival, outcome but, once adjusted for the relevant clinical factors, a significant association between higher β_2_AR expression and improved overall (HR 0.53. 95% CI 0.30–0.94), recurrence‐free (HR 0.57, 95% CI 0.33–0.97) and cancer‐specific survival (HR 0.48, 95% CI 0.27–0.87) was demonstrated compared to lower expression (Table 2). In addition, patients with high β_2_AR status in the second and third tertiles had a significant 70% reduction in the risk of death from any cause (HR 0.30, 95% CI 0.15–0.60 and HR 0.30, 95% CI 0.14–0.61, respectively). Similarly, patients in the second and third tertiles had improved recurrence‐free survival (HR 0.50, 95% CI 0.26–0.97 and HR 0.42, 95% CI 0.22–0.82) and cancer‐specific survival (HR 0.29, 95% CI 0.14–0.60 and HR 0.25, 95% CI 0.11–0.54). Following Kaplan–Meier analysis, high expression of β_2_AR was also associated with improved survival (log‐rank test *p* = 0.01) (Figure 3).

**Table 2:** Overall survival, cancer‐specific survival, and recurrence‐free survival according to β_2_AR expression

Biomarker	Recurrence‐free survival				Overall survival^†^				Cancer‐specific survival			
	Events	Patients	Unadjusted HR (95% CI)	Adjusted HR* (95% CI)	Events	Patients	Unadjusted HR (95% CI)	Adjusted HR* (95% CI)	Events	Patients	Unadjusted HR (95% CI)	Adjusted HR* (95% CI)
β_2_ adrenergic receptor (based on maximum H‐scores)												
Low (<180, median)	37	55	1.00	1.00	36	56	1.00	1.00	35	55	1.00	1.00
High (≥180, median)	37	64	0.72 (0.45–1.13)	0.57 (0.33–0.97)	34	66	0.68 (0.43–1.09)	0.53 (0.30–0.94)	30	62	0.65 (0.40–1.05)	0.48 (0.27–0.87)
Tertile 1 (<140)	33	46	1.00	1.00	33	47	1.00	1.00	32	46	1.00	1.00
Tertile 2 (<140–270)	21	37	0.72 (0.42–1.25)	0.50 (0.26–0.97)	19	38	0.62 (0.35–1.09)	0.30 (0.15–0.60)	17	36	0.61 (0.34–1.09)	0.29 (0.14–0.60)
Tertile 3 (>270)	20	36	0.64 (0.36–1.11)	0.42 (0.22–0.82)	18	37	0.57 (0.32–1.01)	0.30 (0.14–0.61)	16	35	0.52 (0.29–0.95)	0.25 (0.11–0.54)

CI, confidence interval; HR, hazard ratio.

* Adjusted for age at diagnosis, sex, nodal status, grade, PET response, circumferential resection margin status, lymphatic vascular invasion, primary site, and smoking.

^†^ This analysis included 117 patients as 5 had died due to other causes.

### Sensitivity analysis

Considering the differing molecular biology between oesophageal and GOJ tumours, a sensitivity analysis was conducted by restricting the patient cohort to patients with GOJ tumours. In keeping with the results for the whole cohort, there were no significant associations with β_1_AR expression and any survival outcome (supplementary material, Table S4). Higher β_2_AR expression was associated with improved recurrence‐free, overall, and cancer‐specific survival, and the magnitude of associations were marginally strengthened (adjusted HR 0.53, 95% CI 0.29–0.96; HR 0.50, 95% CI 0.27–0.94; HR 0.45, 95% CI 0.23–0.87, respectively). Specially, in the highest expression tertile, β_2_AR was associated with markedly improved overall (HR 0.27, 95% CI 0.13–0.59) and cancer‐specific survival (HR 0.22, 95% CI 0.09–0.52) (Table 3).

**Table 3:** Overall survival, cancer‐specific survival, and recurrence‐free survival according to β_2_‐adrenergic receptor expression restricting to patients with gastro‐oesophageal junction tumours

Biomarker	Recurrence‐free survival				Overall survival				Cancer‐specific survival			
	Events	Patients	Unadjusted HR (95% CI)	Adjusted HR* (95% CI)	Events	Patients	Unadjusted HR (95% CI)	Adjusted HR* (95% CI)	Events	Patients	Unadjusted HR (95% CI)	Adjusted HR* (95% CI)
β_2_ adrenergic receptor (based on maximum H‐scores)												
Low (<180, median)	33	47	1.00	1.00	32	48	1.00	1.00	31	47	1.00	1.00
High (≥180, median)	31	52	0.72 (0.44–1.18)	0.53 (0.29–0.96)	30	54	0.73 (0.44–1.20)	0.50 (0.27–0.94)	26	50	0.69 (0.41–1.17)	0.45 (0.23–0.87)
Tertile 1 (<140)	29	39	1.00	1.00	29	40	1.00	1.00	28	39	1.00	1.00
Tertile 2 (<140–270)	19	30	0.85 (0.48–1.52)	0.52 (0.25–1.09)	17	31	0.72 (0.39–1.31)	0.27 (0.12–0.58)	15	29	0.72 (0.39–1.36)	0.25 (0.10–0.58)
Tertile 3 (>270)	16	30	0.60 (0.32–1.10)	0.41 (0.20–0.83)	16	31	0.59 (0.32–1.08)	0.27 (0.13–0.59)	14	29	0.53 (0.28–1.01)	0.22 (0.09–0.52)

CI, confidence interval; HR, hazard ratio.

* Adjusted for age at diagnosis, sex, nodal status, grade, PET response, circumferential resection margin status, lymphatic vascular invasion, and smoking.

## Discussion

In this study we investigated the association between the expression of the beta‐adrenergic receptors (β_1_ and β_2_AR) and survival outcomes in resectable OAC patients. High β_2_AR expression was associated with significantly improved recurrence‐free, overall and cancer‐specific survival rates, which was particularly evident in patients with GOJ tumours. These findings highlight the potential prognostic value of β_2_AR expression in predicting not only the risk of death but also the likelihood of disease recurrence.

To our knowledge, this is the first study to analyse βAR expression and survival in OAC patients. Several studies observed different cancer types and have demonstrated conflicting results. In colorectal adenocarcinoma, high β_2_AR expression was associated with worse prognosis, with poor disease‐free (HR 3.17; 95% CI 2.29–9.68; *p* < 0.01) and overall survival (HR 2.27; 95% CI 1.51–3.72; *p* < 0.01), as well as increased tumour aggressiveness, cell proliferation and metastasis in a cohort of 147 patients in Japan who underwent surgical resection [8]. In a study of 278 oestrogen receptor‐negative breast cancer patients in Japan, high expression of β_2_AR was also associated with poor cancer‐specific survival (HR 2.53; 95% CI 1.15–5.58; *p* = 0.021) and in a further study in Japan among 133 patients with resected malignant melanoma, high β_2_AR was associated with increased tumour thickness (*p* < 0.001), ulceration (*p* = 0.002), advanced stage (T stage, *p* < 0.001; N stage, *p* = 0.015), and poor overall survival (HR 1.69; 95% CI 1.2–2.45; *p* = 0.002) [11, 23]. In contrast, in a study of 202 HER2+ breast cancer patients in Belgium, high β_2_AR was associated with improved disease‐free survival (HR 0.52; 95% CI 0.32–0.84; *p* = 0.0068), decreased expression of angiogenesis and proliferation‐related genes and increased immune mediators [12]. Another study in 106 patients with surgically resected oral squamous cell carcinoma in Brazil demonstrated that patients with high β_2_AR expression had improved overall and cancer‐specific survival [24]. Therefore, the prognostic significance of β_2_AR expression likely differs by cancer subtype.

Recent evidence has shed light on the potential mechanism of the prognostic effect of β_2_AR signalling through its influence on cancer progression by regulating cell proliferation, dysregulation of DNA repair and increasing cell invasion and metastasis [25, 26, 27]. Stress‐induced signalling through βARs appears to produce an immunosuppressive tumour micro‐environment in response to chronic exposure to catecholamines [28]. β_2_AR activation suppresses immune surveillance by reducing the proliferation and activation of CD8+ T cells, leading to T cell exhaustion, and driving macrophages towards a tumour‐promoting M2 phenotype [29, 30, 31, 32]. Combined with a decrease in pro‐inflammatory cytokine production by macrophages and monocytes as well as increased populations of regulatory T cells and myeloid‐derived suppressor cells, these factors all establish a pro‐tumoural immunophenotype [33, 34]. β_1_AR signalling has recently been implicated as an immune checkpoint capable of inducing T cell exhaustion leading to impairment of their proliferation and cytokine production [13]. Indeed, pharmacological blockade of both β_1_AR and β_2_AR has been associated with improved overall survival in patients with metastatic melanoma treated with immune‐checkpoint inhibitors [35]. This effect has primarily been attributed to inhibition of β_2_AR signalling as the survival benefit appears to be lost in patients taking β_1_‐selective‐β‐blockers, supporting the ability of non‐selective β‐blockers to reverse the immunosuppressive, cancer‐promoting phenotype induced by βAR signalling [36]. However, a recent trial of adjuvant immune checkpoint blockade of programmed cell death protein 1 (PD‐1) in malignant melanoma demonstrated an improved recurrence‐free survival in patients taking concurrent β_1_‐selective‐β‐blockers, indicating the complex role both receptors may play in influencing the efficacy of immunotherapy and cancer prognosis in general [37].

To date, few studies have investigated the role of βAR signalling in oesophageal cancer. A report from our group examined the risk of oesophageal cancer in a large, nested case–control study in 1,979 oesophageal cancer patients matched to 9,543 controls from the Scottish Primary Care Clinical Informatics Unit (PCCIU) database, with a separate cohort study conducted within the UK Biobank among 476,123 participants (of whom 355 developed oesophageal cancer), to investigate the role of a number of oesophageal sphincter relaxing medications, including β_2_ agonists. Use of β_2_ agonists was associated with an increased risk of developing oesophageal cancer compared to non‐use (OR 1.38, 95% CI 1.12–1.70) in the PCCIU database; however, no association was demonstrated in the UK Biobank for oesophageal cancer or OAC specifically [38]. In contrast, our data show that high β_2_AR expression is a marker of improved prognosis in resected OAC, whereas β_1_AR expression has no prognostic effect. In addition, β_2_AR was suggested to have a stronger influence on survival in GOJ tumours compared to those of the lower third of the oesophagus. These findings may be the result of the effects of neoadjuvant chemotherapy in our cohort, where β_2_AR expression may be increased in patients in whom neoadjuvant chemotherapy has been most effective. Also, the differing molecular biology between oesophageal and GOJ tumours, with the former dominated by the TCGA chromosomal instability subtype, may account for the stronger association with survival observed when the analysis was restricted to junctional tumours [39]. This biological difference is supported by the presence of β_2_AR in the gastrointestinal tract, and gastric tissue in particular, whereas β_1_AR is predominantly expressed in cardia tissue with low expression in the gut [40]. This tissue specific distribution of the βARs suggests that each receptor subtype may play a different role depending on the tumour site.

Our study has several strengths including the population‐based design and accurate reflection of current practice, as our cohort of patients have undergone neoadjuvant chemotherapy. With regard to limitations, the samples obtained may be subject to centre‐specific technical factors, and so a multi‐centre validation in a suitably powered cohort would be essential to further investigate β_2_AR as a prognostic marker in OAC. The scoring of β_1_AR was also impacted by a large proportion of strongly staining samples leading to a high median value and imbalance in the number of patients scoring as high and low. This likely reflects our scoring methodology of using divisions of 10% for the percentage of stained tumour cells but also indicates that this marker may be challenging to implement clinically due to its predominantly strong staining in a high percentage of tumour cells and a lack of variation. Our study is also limited by an absence of data on the use of β‐blockers in our patient population. Finally, our cohort represents patients with relatively good prognosis due to the presence of surgically resectable disease, and so we cannot deduce the role of β_2_AR expression as a prognostic marker in more advanced disease.

In conclusion, we have identified that higher β_2_AR expression is associated with improved survival for OAC patients treated with neoadjuvant chemotherapy followed by surgical resection. Use of prognostic markers following resection of OAC has the potential to inform surveillance strategies and identify patients at high risk of relapse, allowing early treatment intervention. Our results also provide the basis for future studies on the potential of β_2_AR expression modulation as a therapeutic target. Further research is warranted to clarify the underlying mechanisms by which β_2_AR contributes to improved patient outcomes and to explore its potential as a prognostic marker in OAC.

## Author contributions statement

ÚCM and RCT designed the study and interpreted data. TO, SS, JT, DTM, SM, ÚCM and RCT carried out data collection. Experimental work was performed by TO, SS, JT, DTM, SM and CL. JAJ, TO, SS, JT, DTM, SM, HGC, ÚCM and RCT analysed the data. All authors were involved in writing the manuscript and had final approval of the submitted and published versions.

## Supporting information


**Figure S1.** Flowchart depicting the patient selection process
**Figure S2.** Violin plot of β‐adrenergic receptors scores' distribution
**Figure S3.** Kaplan–Meier plot of 5‐year cancer‐specific survival rates and β_1_AR expression
**Table S1.** REMARK checklist
**Table S2.** Characteristics of eligible patients included in the study
**Table S3.** Recurrence‐free, overall survival, and cancer‐specific survival according to β_1_AR expression
**Table S4.** Recurrence‐free, overall, and cancer‐specific survival according to β_1_AR expression restricting to patients with gastro‐oesophageal junction tumours

## Data Availability

All quantitative data are available upon request to the corresponding author.
