# Supplementary material for: Prognostic significance of beta‐adrenergic receptor expression in oesophageal adenocarcinoma
Source: J Pathol Clin Res. 2026 Jan 4;12(1):e70070. doi: 10.1002/2056-4538.70070 (PMC12765628; doi:10.1002/2056-4538.70070)
Supplement: Supplementary file 1 — Figure S1. Flowchart depicting the patient selection process Figure S2. Violin plot of β‐adrenergic receptors scores' distribution Figure S3. Kaplan–Meier plot of 5‐year cancer‐specific survival rates and β1AR expression Table S1. REMARK checklist Table S2. Characteristics of eligible patients included in the study Table S3. Recurrence‐free, overall survival, and cancer‐specific survival according to β1AR expression Table S4. Recurrence‐free, overall, and cancer‐specific survival according to β1AR expression restricting to patients with gastro‐oesophageal junction tumours [file CJP2-12-e70070-s001.pdf]

Prognostic significance of beta-adrenergic receptor expression in oesophageal adenocarcinoma

T Oliveira *et al*. *J Pathol Clin Res* https://doi.org/10.1002/2056-4538.70070

Supplementary Figures S1–S3

Supplementary Tables S1–S4

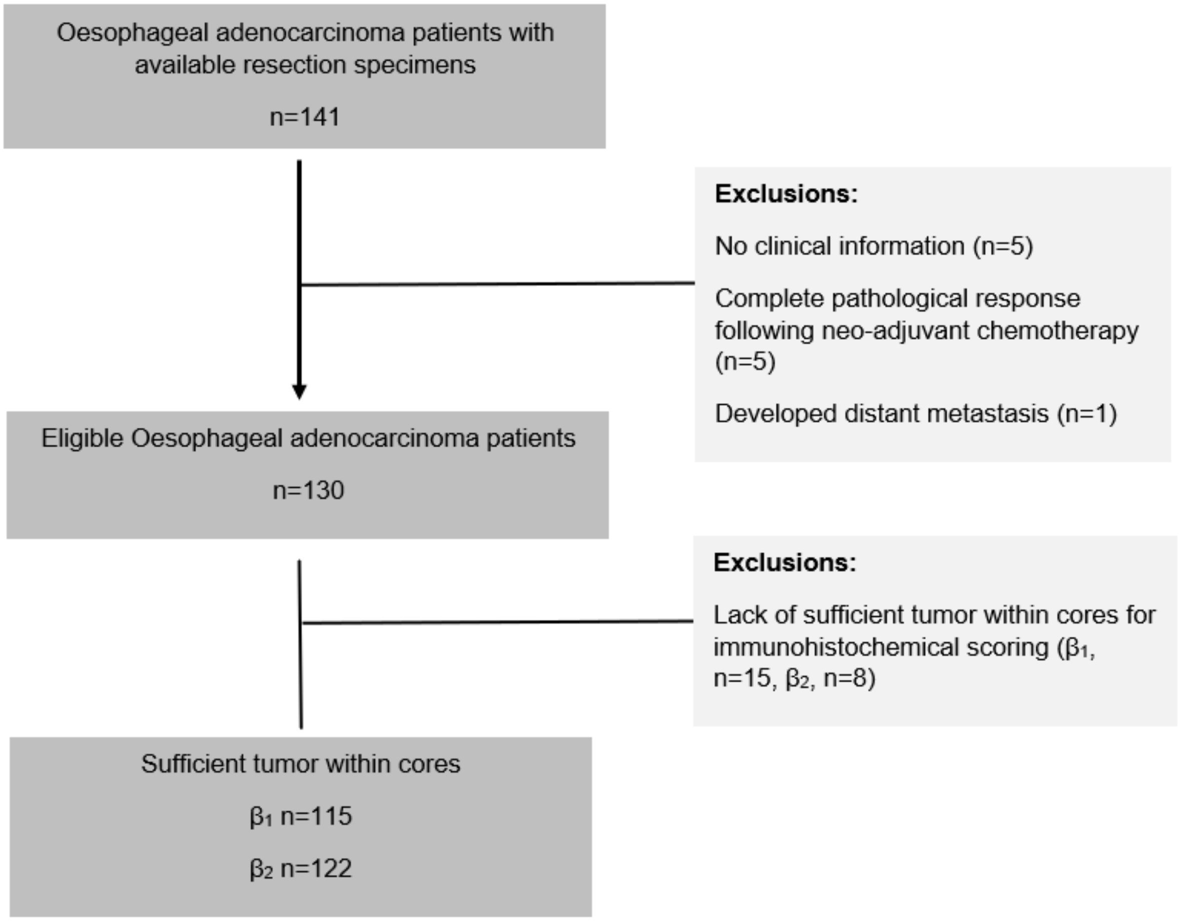

Figure S1. Flowchart depicting the patient selection process.

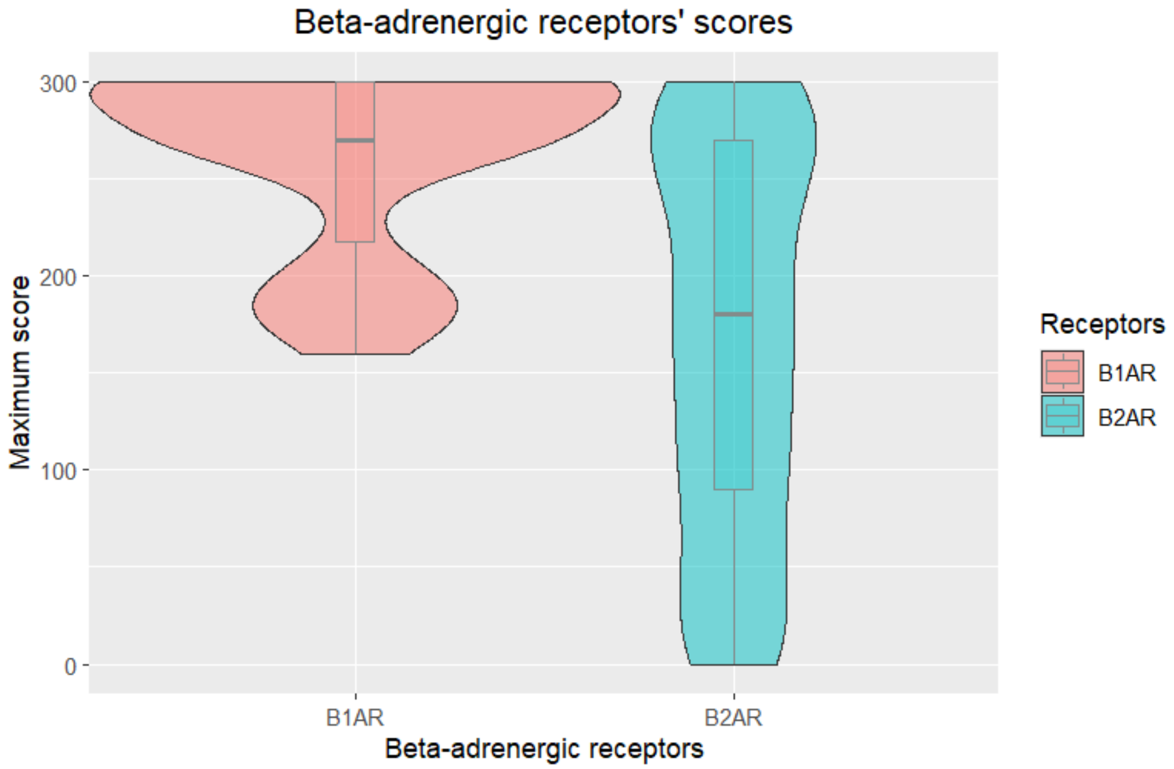

Figure S2. Violin plot of β-adrenergic receptors scores' distribution. The figure shows a violin plot with included boxplot of the median score of the overall maximum scores of each group – 270 and 180 for β_1AR and β_2AR, respectively.

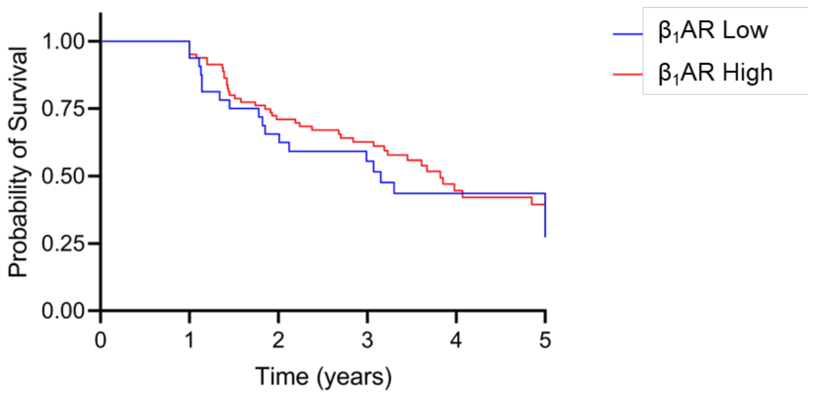

Figure S3. Kaplan–Meier plot of 5-year cancer-specific survival rates and β_1AR expression. No statistically significant results were obtained for cancer-specific survival analysis, or recurrence-free survival (data not shown).

Table S1. REMARK checklist

**INTRODUCTION**		
1	State the marker examined, the study objectives, and any pre-specified hypotheses.	'Introduction' section (page 7)
**MATERIALS AND METHODS**		
Patients		
2	Describe the characteristics (for example, disease stage or co-morbidities) of the study patients, including their source and inclusion and exclusion criteria.	'Study design and cohort' section (page 7) and 'Clinical variables' section (page 9)
3	Describe treatments received and how chosen (for example, randomized or rule-based).	'Study design and cohort' section (page 7)
Specimen characteristics		
4	Describe type of biological material used (including control samples) and methods of preservation and storage.	'Study design and cohort' section (page 7)
Assay methods		
5	Specify the assay method used and provide (or reference) a detailed protocol, including specific reagents or kits used, quality control procedures, reproducibility assessments, quantitation methods, and scoring and reporting protocols. Specify whether and how assays were performed blinded to the study endpoint.	'Immunohistochemistry' section (page 8) and 'Scoring Methods' section (page 8)
Study design		
6	State the method of case selection, including whether prospective or retrospective and whether stratification or matching (for example, by stage of disease or age) was used. Specify the time period from which cases were taken, the end of the follow-up period, and the median follow-up time.	'Study design and cohort' section (page 7)
7	Precisely define all clinical endpoints examined.	'Study design and cohort' section (page 7)
8	List all candidate variables initially examined or considered for inclusion in models.	'Clinical variables' section (page 9) and 'Statistical Analysis' section (page 9)
9	Give rationale for sample size; if the study was designed to detect a specified effect size, give the target power and effect size.	All OAC patients undergoing neoadjuvant chemotherapy and surgical resection in Northern Ireland between 20024 and 2012 were included in the analysis
Statistical analysis methods		
10	Specify all statistical methods, including details of any variable selection procedures and other model-building issues, how model assumptions were verified, and how missing data were handled.	'Statistical Analysis' section (page 9) and 'Patient Demographics and Immunohistochemical Staining' section (page 10)
11	Clarify how marker values were handled in the analyses; if relevant, describe methods used for cutpoint determination.	'Scoring Methods' section (page 8) and 'Patient Demographics and Immunohistochemical Staining' section (page 10)
**RESULTS**		
Data		
12	Describe the flow of patients through the study, including the number of patients included in each stage of the analysis (a diagram may be helpful) and reasons for dropout. Specifically, both overall and for each subgroup extensively examined report the number of patients and the number of events.	'Patient Demographics and Immunohistochemical Staining' section (page 10) and Supplementary figure 1
13	Report distributions of basic demographic characteristics (at least age and sex), standard (disease-specific) prognostic variables, and tumour marker, including numbers of missing values.	'Patient Demographics and Immunohistochemical Staining' section (page 10) and Table 1
Analysis and presentation		
14	Show the relation of the marker to standard prognostic variables.	Table 1
15	Present univariable analyses showing the relation between the marker and outcome, with the estimated effect (for example, hazard ratio and survival probability). Preferably provide similar analyses for all other variables being analysed. For the effect of a tumour marker on a time-to-event outcome, a Kaplan-Meier plot is recommended.	'Patient Demographics and Immunohistochemical Staining' section (page 10), 'β_1AR/β_2AR expression and survival outcomes'

		section (page 11), Table 1 and Figure 3
16	For key multivariable analyses, report estimated effects (for example, hazard ratio) with confidence intervals for the marker and, at least for the final model, all other variables in the model.	'β_1AR/β_2AR expression and survival outcomes' section (page 11) and Tables 1-3
17	Among reported results, provide estimated effects with confidence intervals from an analysis in which the marker and standard prognostic variables are included, regardless of their statistical significance.	Tables 1-3 and Supplementary tables 3 and 4
18	If done, report results of further investigations, such as checking assumptions, sensitivity analyses, and internal validation.	'Sensitivity Analysis' section (page 12, Table 3 and Supplementary table 4
**DISCUSSION**		
19	Interpret the results in the context of the pre-specified hypotheses and other relevant studies; include a discussion of limitations of the study.	'Discussion' section (page 12)
20	Discuss implications for future research and clinical value.	'Discussion' section (page 12)

Table S2. Characteristics of eligible patients included in the study

	Eligible OAC patients for the study
	***n* = 130**
**Sex**	
Male	99 (76.2)
Female	31 (23.8)
**Age at diagnosis (years)**	
<50	11 (8.5)
50-59	27 (20.8)
60-69	62 (47.7)
≥70	30 (23.0)
**Smoking status**	
Non-smoker	31 (23.8)
Ex-smoker	52 (40.0)
Current smoker	31 (23.8)
Unknown	16 (12.4)
**Alcohol**	
Non-drinker	56 (43.1)
Drinker	67 (51.5)
Unknown	7 (5.4)
**Primary tumour site**	
Lower third	22 (16.9)
Gastro-oesophageal junction	108 (83.1)
**Siewert classification**[a]	
Siewert I	69 (63.9)
Siewert II/III	39 (36.1)
**PET response**	
No	44 (33.8)
Yes	65 (50.0)
Unknown	21 (16.2)
**Lymphatic vascular invasion**	
No	47 (36.1)
Yes	82 (63.1)
Unknown	1 (0.8)
**Grade**	
Well or moderate	57 (43.9)
Poor	73 (56.1)
**Circumferential resection margin status**	
Negative	75 (57.7)
Positive	55 (42.3)
Unknown	0 (0.0)
**Surgical T stage**	
1	15 (11.5)
2	26 (20.0)
3	84 (64.6)
4	5 (3.9)
**Surgical N stage**	
0	48 (36.9)
1	26 (20.0)
2	26 (20.0)
3	30 (23.1)

[a]Restricted to patients with gastro-oesophageal junction tumours, T = tumour, N = nodal.

Table S3. Recurrence-free, overall survival, and cancer-specific survival according to β_1-AR expression

Biomarker	Recurrence-free survival				Overall survival				Cancer-specific survival[b]			
	Events	Patients	Unadjusted HR (95% CI)	Adjusted HR[a] (95% CI)	Events	Patients	Unadjusted HR (95% CI)	Adjusted HR[a] (95% CI)	Events	Patients	Unadjusted HR (95% CI)	Adjusted HR[a] (95% CI)
**β_1 adrenergic receptor** (based on maximum H-scores)												
Low (<median, 270)	21	38	1.00	1.00	21	40	1.00	1.00	19	38	1.00	1.00
High (≥median, 270)	47	74	1.20 (0.72, 2.01)	1.21 (0.65, 2.25)	43	75	1.08 (0.64, 1.82)	1.18 (0.63, 2.23)	40	72	1.07 (0.62, 1.84)	1.14 (0.59, 2.19)

CI, confidence interval; HR, hazard ratio.
[a]Adjusted for age at diagnosis, sex, nodal status, grade, PET response, circumferential resection margin status, lymphatic vascular invasion, primary site, and smoking.
[b]This analysis included 117 patients as 5 had died due to other causes.

Table S4. Recurrence-free, overall, and cancer-specific survival according to β1AR expression restricting to patients with gastro-oesophageal junction tumours

Biomarker	Recurrence-free survival				Overall survival				Cancer-specific survival			
	**Events**	**Patients**	**Unadjusted HR (95% CI)**	**Adjusted HR[a] (95% CI)**	**Events**	**Patients**	**Unadjusted HR (95% CI)**	**Adjusted HR[a] (95% CI)**	**Events**	**Patients**	**Unadjusted HR (95% CI)**	**Adjusted HR[a] (95% CI)**
**β_1 adrenergic receptor**												
Low (<median, 270)	16	30	1.00	1.00	17	32	1.00	1.00	15	30	1.00	1.00
High (≥median, 270)	43	64	1.34 (0.75, 2.38)	1.09 (0.54, 2.18)	40	65	1.13 (0.64, 1.99)	1.09 (0.55, 2.19)	37	62	1.12 (0.61, 2.04)	1.00 (0.49, 2.06)

CI, confidence interval; HR, hazard ratio.

[a]Adjusted for age at diagnosis, sex, nodal status, grade, PET response, circumferential resection margin status, lymphatic vascular invasion, and smoking.
